# Recent advances in ankylosing spondylitis: understanding the disease and management

**DOI:** 10.12688/f1000research.14956.1

**Published:** 2018-09-21

**Authors:** Leticia Garcia-Montoya, Hanna Gul, Paul Emery

**Affiliations:** 1 Leeds Institute of Rheumatic and Musculoskeletal Medicine, University of Leeds, Chapel Allerton Hospital, Chapeltown Road, Leeds, LS7 4SA, UK; 2 NIHR Leeds Musculoskeletal Biomedical Research Centre, Leeds Teaching Hospitals NHS Trust, Leeds, UK

**Keywords:** Ankylosing spondylitis, spondyloarthritis, spondyloarthropathy, management, pathogenesis

## Abstract

The term spondyloarthritis refers to a group of immune-mediated diseases characterised by inflammation of the axial skeleton, peripheral joints, and entheses. Ankylosing spondylitis (AS) is the most common and characteristic of these entities and even though it was first described over two centuries ago, the understanding of the underlying disease mechanism remains incomplete. It is known that around 40% of patients with AS have subclinical bowel inflammation, suggesting that the origin of the disease could be in the gut. Also, more genes and new molecules have demonstrated a role in the pathogenesis of AS. In this review, we analyse the latest therapies for spondyloarthritis and the most relevant discoveries over the last three years, together with their implications for different aspects of the disease.

## Introduction

Ankylosing spondylitis (AS) is a chronic immune-mediated inflammatory arthritis included in the so-called group of spondyloarthritis (SpA). It typically develops in males in their third decade of life and affects mainly the axial skeleton and the sacroiliac joints. Although the oldest descriptions date from the time of Galen, it was not until the 19th century that the disease could be accurately diagnosed on the basis of reports by Vladimir Bekhterev, Adolph Strümpell, and Pierre Marie. The HLA-B27 allele is known to have a strong association with the disease ^1^; however, other genes play a part in its development. The discovery of several inflammatory pathways led to the era of the biologic therapies, which meant a revolution in the treatment and prognosis of AS. Tumour necrosis factor inhibitors (TNFis) were the first ones to be approved, but in the last few years the interleukin-17 (IL-17)/IL-23 axis has gained relevance, culminating in the licence of new biological disease-modifying antirheumatic drugs (bDMARDs) blocking IL-17.

In spite of all of these advances, the disease mechanisms underlying the disease are not fully understood and new information concerning the pathogenesis, triggers, and the outcome of new treatments continues to appear. In this review, we will focus on the advances of the last three years regarding the above aspects of AS.

## Genetics

AS is considered an inherited disease, as over 90% of the risk for its development relies on genes ^2^. However, the HLA-B27 allele accounts for only 20% of the genetic effect ^1^. Other alleles, especially HLA-B, are thought to play an important role in the disease: HLA-B*13:02, HLA-B*40:01, HLA-B*47, and HLA-B*51 are some examples ^3^. The most significant discovery of the last three years has been the interaction of ERAP1, the protein endoplasmic reticulum aminopeptidase 1, with the HLA-B alleles, resulting in a higher risk of developing AS. The main variant of the gene (rs30187, K528R) interacts only with the HLA-B27 allele, and in patients who are HLA-B27 negative, ERAP1 interacts with the HLA-B40 allele ^4^. The mechanism underlying the increased risk remains unclear; nevertheless, it is known that the presence of this gene is not related to the radiographic severity of the disease ^5^.

A recent study reported that a lower copy number of the *TLR7* gene was a marker for susceptibility to AS in males but behaves as a protective factor in women ^6^. Ruan *et al*. ^7^ found that genetic polymorphisms of IL-12B (rs6871626) and IL-6R (rs4129267) were associated with an increased risk of AS independently of gender and also could function as biomarkers for diagnosis and prognosis.

Genome-wide association studies have shown that the T helper 17/23 (Th17/23) axis and its multiple genetic polymorphisms are involved not only in AS but also in inflammatory bowel disease (IBD) and psoriasis, supporting the hypothesis that there is a common underlying pathogenic mechanism and that the microbiome seems to be implicated in the development of the diseases ^8^.

## Pathogenesis

How HLA-B27 initiates AS is unknown, and, after many years, some of the earliest hypotheses are still being investigated. The original hypothesis, called the ‘arthritogenic peptide theory’, suggests that the presentation of either bacterial peptides by HLA-B27 or self-mimicking HLA-B27-binding peptides from certain bacteria could initiate a cell-mediated immune reaction leading to AS ^9^. The second one is the ‘unfolded protein response’ hypothesis, which suggests that HLA-B27 tends to misfold and accumulate in the endoplasmic reticulum, triggering a stress response that results in the release of IL-23 ^10, 11^. However, a new study ^12^ has questioned these two theories, claiming that the arthritogenic peptide theory should be reassessed in terms of quantitative changes concerning self-peptide presentation and T-cell selection. It also stated that the absolute binding preferences of HLA-B27 allotypes are not sufficient to explain the association of the disease.

The third hypothesis is the ‘HLA-B27 homodimer model’ ^13^, which supports the view that HLA-B27 homodimers have an abnormal interaction with natural killer (NK) and CD4 T cells. Unlike the heterodimeric form of HLA-B27, the homodimer is able to bind to certain killer cell immunoglobulin-like receptors (KIRs), which are expressed on NK cells and T cells, causing the release of IL-17 ^14– 16^. Ridley *et al*. ^17^ have proven that CD41 T cells upregulate the expression of KIR-3DL2 on the cell surface and that the binding of this receptor to HLA-B27 potentiates T-cell survival and Th17 cell differentiation. Th17 cells are a type of T-helper lymphocyte which produces IL-17 ^18^, a cytokine able to increase T-cell priming and stimulate immune cells such as fibroblasts and macrophages promoting the release of IL-6, TNF-α, and other chemokines ^19^.

Oppmann *et al*. found that IL-23 is one of the triggers of the Th17 response ^20^. This molecule is a pro-inflammatory cytokine that seems to play an important part in stabilising Th17 cell phenotype through the transcription factor Blimp-1 (Prdm1) ^21^, which is associated with Crohn’s disease ^22^. Th17 cells are commonly found in the intestinal lamina propria of the gut ^23^ and their instability can be induced by exposure to certain bacteria, leading to the transition of Th17 cells into regulatory T (Treg) cells ^24^. Maxwell *et al*. ^25^ also suggested that IL-23 promotes the accumulation of Treg cells in the bowel, some of which probably were Th17 previously ^26, 27^.

## Microbiome

Gut mucosal inflammation is estimated to be present in 70% of patients with AS ^28^, progressing to clinical IBD in 5% of cases. Crohn’s disease and ulcerative colitis are characterised by having gut dysbiosis (a qualitative or quantitative microbial imbalance) which is also seen in AS ^29^. The Ghent inflammatory arthritis and spondylitis cohort (GIANT) studies strongly support the concept that there is a relationship between gut inflammation and the pathogenesis of AS ^30– 32^. One of the theories to explain it is that a constant antigenic stimulation can activate T cells and this might be responsible for chronic bowel inflammation ^33^. Other studies suggest that patients with AS (and their first-degree relatives) have a high gut permeability, which increases their exposure to gut microbes ^34^. In addition, animal studies proved that HLA-B27 alone was insufficient to develop AS, since transgenic rats that had been raised in a germ-free environment did not develop features of SpA ^35^. In the last few years, investigators have focussed on detecting pathogens as triggers for AS. *Klebsiella pneumoniae* was the first to be reported ^36^: this bacterium is thought to carry an antigen that resembles a molecule coded by the *HLA-B27* gene; however, the mechanism is not fully understood and other studies have stated that its involvement in AS is unlikely. Other relevant families of bacteria that have been associated with the development of AS are *Lachnospiraceae*, *Prevotellaceae*, *Rikenellaceae*, *Porphyromonadaceae*, and *Bacteroidaceae*. Non-gut bacteria are also thought to be involved: periodontal disease has become a possible target as anti- *Porphyromonas gingivalis* and anti- *Prevotella intermedia* antibodies are detected in high titres in patients with SpA ^37^. Some studies even suggest that chronic periodontitis is associated with severe spinal dysmobility in AS ^38^. Nevertheless, it is still an area of investigation, as recently published results contradict these findings ^39^.

## Gender differences

Evidence gained in the last few years suggests that AS affects men and women differently. Landi *et al*. ^40^ analysed a sample of 2,044 patients with AS and reported that disease commences earlier in men but that the diagnosis is usually more delayed than in women. Men have lower disease activity measured by Bath Ankylosing Spondylitis Disease Activity Index (BASDAI) and Assessment of SpondyloArthritis international Society (ASAS)-endorsed Disease Activity Score (ASDAS) and a better quality of life (Ankylosing Spondylitis Quality of Life Questionnaire, or ASQol) but have worse spinal mobility (Bath Ankylosing Spondylitis Metrology Index, or BASMI) and a more serious radiologic progression (Bath Ankylosing Spondylitis Radiology Index, or BASRI). In contrast, women usually have more peripheral arthritis and an increased prevalence of arthritis, dactylitis, and enthesitis combined with a worse quality of life and a worse response to anti-TNF treatment ^41^. This contradicts the results reported by Webers *et al*. ^42^, who agreed that men had more radiographic damage and a better quality of life but did not find differences in disease activity or physical function. However, the authors analysed only 216 patients.

## Therapies

Since 2015, there has been remarkable progress concerning the therapeutic management of AS. New treatments and strategies have paralleled the description of new pathogenic mechanisms. Table 1 summarises the most relevant drugs and their mechanism of action; some are still under investigation ^43– 55^.

**Table 1.** Novel approaches for the management of ankylosing spondylitis.

Therapy	Reference	Mechanism of action	Current status
CT-P13 (infliximab biosimilar)	43	Anti-TNF	Approved for use in AS
SB4 (etanercept biosimilar)	44	Anti-TNF	Approved for use in AS
Secukinumab	45	Anti-IL-17A	Approved for use in AS
Ixekizumab	46	Anti-IL-17A	Under investigation. Primary endpoints met.
Brodalumab	47	Anti-IL-17R	Not approved owing to safety issues
Ustekinumab	48, 49	Anti-IL-12/IL-23	Not approved owing to lack of efficacy
ABT-122	50	Anti-IL-17A/TNF-α	Under investigation. Successful phase II clinical trials
COVA322	51	Anti-IL-17A/TNF-α	Not approved owing to safety issues
CBP30	52	CBP/p300 bromodomain inhibition	Future trials announced
Sarilumab	53	Anti-IL-6Rα	Not approved owing to lack of efficacy
Tofacitinib	54	JAK inhibitor	Under investigation. Successful phase II clinical trials

AS, ankylosing spondylitis; IL, interleukin; JAK, Janus kinase; TNF, tumour necrosis factor.

### Tumour necrosis factor inhibitors

The first bDMARDs were approved several years ago. As the patents of some of these drugs were closer to expiration, pharmaceutical companies focused their interest on developing more affordable drugs. CT-P13, the infliximab biosimilar, was the first one to be released ^43^, followed by Benepali (SB4), the etanercept biosimilar, in 2016 ^56^. Multiple trials endorse the similar safety and efficacy of CP-P13 and infliximab not only in patients with AS but also in the treatment of rheumatoid arthritis (RA). The PLANETAS study reported comparable results for patients with AS regarding ASAS20 and ASAS40 ^57^. The extension of the study showed that switching from infliximab to its biosimilar did not imply negative effects on safety or efficacy ^58^. These findings were also confirmed in the PLANETRA study, carried out in patients with RA ^59, 60^. In regard to Benepali (SB4), the most important efficacy and safety studies have been carried out in RA, showing results similar to those obtained with Enbrel ^44^. As far as therapy switching is concerned, the safety and efficacy are maintained after switching from Enbrel to Benepali in patients with RA ^61^, and a study is taking place in Germany to assess this transition in patients with AS ^62^.

### Interleukin inhibitors

The approval of secukinumab, a fully human monoclonal antibody able to neutralise IL-17A, has been a major advance in the treatment of AS ^45^. Patients with AS are known to have high levels of this interleukin ^63^, which is critically involved in the pathogenesis of the disease. IL-23 is secreted by antigen-presenting cells and stimulates Th17 cells, which are defined by their production of IL-17 cytokines ^64^.

Two double-blind, placebo-controlled, phase III clinical trials—MEASURE 1 ^65^ and MEASURE 2 ^66^—reported a significant improvement in disease activity in patients with AS with effects sustained over 2 years; this was combined with a good safety profile. Pavelka *et al*. ^67^ recently published the results of MEASURE 3, a randomised, double-blind, phase III trial in patients with active AS. In accordance with previous studies, a significant improvement was reported in the 16-week follow-up compared with placebo and this was sustained until week 52. The patients initially on placebo, who were randomly assigned on week 16, also experienced improvement through week 52. During this time, no major new adverse events were noted. In addition, MEASURE 5, another randomised, double-blind, phase III trial is ongoing. It is also comparing the safety and tolerability of secukinumab in patients with active AS versus placebo ^68^.

Nevertheless, there are other drugs targeting IL-17. COAST-W ^69^ is a phase III, randomised, double-blind, placebo-controlled trial assessing the effect of ixekizumab on radiographic axial SpA. Ixekizumab is also a fully human monoclonal antibody that binds to IL-17A. The first results show that the drug met all of the primary and secondary endpoints. Brodalumab, a monoclonal antibody neutralising IL-17R, proved its efficacy for psoriatic arthritis; however, development was paused after a higher incidence of suicidal ideation was noted ^47^.

IL-23 is directly related to the development of enthesitis ^70^, and the IL-23/IL-17 axis occupies a central place in the pathogenesis of SpA. Ustekinumab is a human monoclonal antibody targeting the p40 subunit of IL-12 and IL-23. It is considered one of the most effective treatments for psoriasis ^71^; however, its efficacy in AS has not been as expected, and results have been contradictory: TOPAS, a prospective, open-label, single-arm, proof-of-concept clinical trial ^72^, reported a reduction of signs and symptoms in patients with active AS. In contrast, a phase III, multicentre, randomised, double-blind, placebo-controlled study ^48^ evaluating the efficacy and safety of ustekinumab in the treatment of non-radiographic axial SpA had to end prematurely after a related study ^49^ did not achieve the key points.

In regard to future approaches, ABT-122, an immunoglobulin molecule targeting both IL-17A and TNF-α, has recently demonstrated its efficacy in phase I and II trials for RA and psoriatic arthritis ^50, 73^; however, given the importance of IL-17 in the pathogenesis of AS, it is expected that trials in patients with AS will commence soon. COVA322 ^74^ is another dual agent, a fusion protein antibody able to bind TNF-α and IL-17A. It was thought to be a promising therapy for AS; however, owing to safety issues in a trial for psoriatic arthritis, investigations have not gone further ^51^. CBP30 ^52^ is a selective inhibitor of CBP/p300 bromodomains able to suppress the production of cytokines by Th17 cells in patients with AS and healthy controls. Future trials with this molecule have been announced. Sarilumab, a fully human monoclonal antibody that blocks the α-receptor of IL-6, demonstrated a lack of efficacy for the treatment of AS in the ALIGN study ^53^.

### Janus kinase inhibitors

Tofacitinib, a Janus kinase (JAK) inhibitor, is also able to interfere in the inflammatory cascade of IL-17, IL-21, and IL-23. In a 16-week clinical trial (with 12 weeks of treatment and 4 weeks of washout period), van der Heijde *et al*. reported the superiority of the 5 mg dose twice a day versus placebo: 63% and 40% of patients, respectively, achieved ASAS20 after 12 weeks ^54^.

An important aspect for biologic therapies is the cost. As more patients receive bDMARDs, healthcare systems have been forced to find new ways to cope financially: one cost-saving strategy is tapering. The 2016 update of ASAS-European League Against Rheumatism (ASAS-EULAR) recommendations for the management of axial AS ^75^ suggested that patients in sustained remission are candidates to taper the dose of the drug. However, there is controversy regarding this approach because of conflicting results. One single-centre prospective study ^76^ compared the evolution of two groups of patients: one receiving TNFis at the standard dose and another group with down-titration. After one year of treatment, the clinical outcomes were similar in the two arms but costs were significantly reduced in the latter group. Plasencia *et al*. ^77^ previously reported similar results in a retrospective study comparing patients in Spain on tapering strategy versus patients on standard dose from the Netherlands. Even though the proportion of patients who maintained remission was similar, those in the tapering group had more flares than the patients receiving the standard dose. Regarding radiographic progression, Park *et al*. ^78^ found that patients who had syndesmophytes at baseline had a more rapid radiographic progression if they received a tapering regime.

### Other therapies

bDMARDs are not the only therapies that have been developed within the last few years. Fattahi *et al*. ^79^ reported the results of a randomised, placebo-controlled trial of a new non-steroidal anti-inflammatory drug (NSAID): B-D-mannuronic acid. The 12-week ASAS response was similar to that obtained with naproxen, and the safety profile was considerably better; there were no renal side effects and gastrointestinal tolerability was good.

There has also been controversy regarding the administration of NSAIDs. Wanders *et al*. ^80^ reported that continuous administration of NSAIDs reduced radiographic progression in comparison with on-demand therapy, whereas more recently Sieper *et al*. ^81^ found that continuous treatment with diclofenac for 2 years did not reduce radiographic progression compared with an on-demand administration.

## Biomarkers

C-reactive protein (CRP) and erythrocyte sedimentation rate (ESR) are two acute-phase reactants that classically have been used to assess the presence of inflammation in patients. Unfortunately, they are not specific and the sensitivity is also low ^82^, especially in patients with non-radiographic axial SpA ^83^ where acute-phase proteins remain within normal limits most of the time.

Owing to the lack of a reliable test, many studies have tried to find a biologic marker capable of predicting a clinical outcome or disease activity in AS. After the discovery of new pathological pathways, interleukins became targets. IL-6 was in the spotlight for some years; but, whereas some studies reported an association between IL-6 and disease activity ^84– 86^, others produced opposite results ^87, 88^.

Calprotectin is a dimer of calcium-binding proteins used as a surrogate marker for gut inflammation. Its main application is monitoring disease activity in IBD ^89^. Both Crohn’s disease and ulcerative colitis are known to be associated with AS. The pathway is not clear; there are discrepancies concerning whether gut inflammation is the cause or the consequence of musculoskeletal disease. Given this, studies have tried to evaluate calprotectin for the management of AS. Duran *et al*. ^90^ found that faecal calprotectin was associated with a higher disease activity and is a better predictor of bowel involvement than CRP, ESR, BASDAI, and Bath Ankylosing Spondylitis Functional Index (BASFI). Klingberg *et al*. ^91^ confirmed these results and also suggested that calprotectin could be used to identify patients with AS at high risk of developing IBD. In accordance with this, another study ^92^ demonstrated that exercise could decrease the levels of calprotectin in patients with AS and that this was associated with a reduction of disease activity.

## Bone involvement

A typical feature of AS is bone formation co-existing with bone resorption. As with many other inflammatory diseases, there is an imbalance between these processes, which results in the appearance of syndesmophytes and bone erosions in patients with AS ^93^. IL-17, one of the most active cytokines in SpA, has a major role promoting osteoclastogenesis directly and through the activation of receptor activator of nuclear factor kappa B (RANK) ^94, 95^. However, it does not exert its action alone; it works in combination with TNF-α. The latter molecule triggers bone destruction through the RANK-RANK ligand (RANK-RANKL) system and inhibits bone formation via the overexpression of Dickkopf-related protein 1 (DKK1) ^96^, which suppresses the WNT bone pathway ^96, 97^. WNT/b-catenin signalling is a regulator of osteogenesis and its high levels promote the formation of osteoblasts and reduce osteoclastogenesis (and therefore bone resorption) ^98^. In contrast to TNF-α, IL-17 has a dual effect because it can promote not only bone destruction by acting complementarily with TNF-α ^99^ but also bone formation at sites of inflammation or exposed to mechanical stress ^100^. An international study ^101^ reported contradictory results, as they found that patients with AS had decreased indicators of osteoclast formation and bone resorption, suggesting a reduced capacity of osteoclast precursors to differentiate into osteoclasts and resorb, probably because of low RANKL/OPG and CD51/CD61 expression.

Ranganathan *et al*. ^102^ found that macrophage migration inhibitory factor (MIF) was able to act on osteoblasts, promoting inflammation and new bone formation. They also suggested that the main source of MIF-producing cells was in the gut and that certain pathogens can induce its release. In addition, the level of MIF was predictive of progressive spinal damage.

More cytokines are being implicated in the pathogenesis of AS. El-Zayadi *et al*. ^103^ recently reported that IL-22 regulates the function of mesenchymal cells, inducing proliferation, migration, and osteogenesis, in an inflammation-dependent context. IL-32γ has been shown to be elevated in the joints and tissues of patients with AS and is able to induce osteoblast differentiation and atypical new bone formation ^104^. Another interleukin, IL-37, is also elevated in patients with osteoporosis in addition to AS, and its levels correlate with disease activity and bone mineral density ^105^. This is consistent with Kwon *et al*. ^106^, who found that osteoblast-lineage cells are increased in patients with AS and are reduced after therapy with infliximab. They also reported that the drug allows mature osteoblast differentiation in late inflammation.

Machado *et al*. ^107^ stated that fat deposition in the vertebral corners is associated with radiographic progression, and they postulated that there should be a ‘window of opportunity’, as fat lesions can be preceded by inflammatory lesions. Interestingly, they also found that even though the absence of bone marrow oedema and fat deposition was negatively associated with radiographic progression, there was still bone formation in those areas, implying that there must be other pathways stimulating osteoproliferation.

## Imaging

The assessment of structural damage is valuable in patients with AS. The modified Stoke Ankylosing Spondylitis Spinal Score (mSASSS) (which consists of the sum of scores of the lumbar and cervical spine from a lateral view, ranging from 0 to 72) is a commonly used method for detecting changes in plain radiographs ^108^; however, X-rays are not a very sensitive tool and they are not able to properly differentiate squaring of vertebral bodies. Kim *et al*. ^109^ evaluated the squaring of the first sacral vertebra (S1) by using a method proposed by Ralston *et al*. ^110^: squaring was associated with mSASSS and changes in magnetic resonance imaging (MRI) and therefore suggested that this assessment could be used to predict early axial involvement of the spine in AS. Regarding other predictors of progression, Dougados *et al*. ^55^ found that HLA-B27-negative patients with normal CRP and normal MRI of the sacroiliac joints have a 1.2% likelihood of progressing to radiological axial SpA within 5 years. This likelihood of progression increased to 18.4% in HLA-B27-positive patients, with raised CRP and bone marrow oedema in the MRI of the sacroiliac joints. In HLA-B27-positive patients, smoking also seems to be associated with a proliferative metabolic pattern of the cartilage, which could justify its outgrowth and the development of enthesopathic lesions ^111^.

Althoff *et al*. were the first to suggest that contrast was not needed to assess axial activity in AS ^112^. However, Zhao *et al*. ^113^ confirmed these results by using a large sample of patients, showing that the combination of short tau inversion recovery (STIR) and diffusion weighted imaging (DWI) in MRI is sufficient to monitor disease activity, avoiding the unnecessary use of contrast with its risks. Conversely, Bradbury *et al*. ^114^ found that DWI has a moderate utility for assessing disease activity or monitoring response to treatment; however, it seems to be a great tool to distinguish axial SpA and non-inflammatory back pain.

Another MRI finding has been the discovery of the ‘backfill sign’. It was initially described by Weber *et al*. ^115^, who defined it as a high signal intensity filling the sacroiliac joint space on T1-weighted images which could represent fat metaplastic tissue filling excavations in subchondral bone; however, the histopathological origin has not been assessed yet. Recently, studies confirmed that the ‘backfill sign’ had a high specificity for SpA (between 95.8% and 98%) ^116^ but the sensitivity was only 59% ^117^. Therefore, its presence could be used to support the diagnosis of axial SpA, but its absence should not be used to exclude it.

## Summary

The numerous investigations and studies carried out within the last three years have improved the understanding of the pathogenesis of AS. This has facilitated the development of new treatment strategies with the consequent improvement of the quality life of patients with SpA. As more is known about the disease, the greater the complexity that is revealed, emphasising the need to continue investigation to achieve even more efficient control of the disease.

## References

[1] Chen B Li J He C: Role of HLA-B27 in the pathogenesis of ankylosing spondylitis (Review). *Mol Med Rep.* 2017;15(4):1943–51. 10.3892/mmr.2017.6248 28259985 PMC5364987

[2] Reveille JD: The genetic basis of ankylosing spondylitis. *Curr Opin Rheumatol.* 2006;18(4):332–41. 10.1097/01.bor.0000231899.81677.04 16763451

[3] Rubin LA Amos CI Wade JA: Investigating the genetic basis for ankylosing spondylitis. Linkage studies with the major histocompatibility complex region. *Arthritis Rheum.* 1994;37(8):1212–20. 10.1002/art.1780370816 8053961

[4] Cortes A Pulit SL Leo PJ: Major histocompatibility complex associations of ankylosing spondylitis are complex and involve further epistasis with *ERAP1*. *Nat Commun.* 2015;6: 7146. 10.1038/ncomms8146 25994336 PMC4443427

[5] Ozen G Deniz R Eren F: Association of *ERAP1*, *IL23R* and *PTGER4* Polymorphisms with Radiographic Severity of Ankylosing Spondylitis. *Open Rheumatol J.* 2017;11:1–9. 10.2174/1874312901711010001 28400866 PMC5366379

[6] Wang M Xu S Zhang X: Association of TLR7 gene copy number variations with ankylosing spondylitis in a Chinese population: a case control study. *Clin Exp Rheumatol.* 2018. 29533758

[7] Ruan WF Xie JT Jin Q: The Diagnostic and Prognostic Role of Interleukin 12B and Interleukin 6R Gene Polymorphism in Patients With Ankylosing Spondylitis. *J Clin Rheumatol.* 2018;24(1):18–24. 29200018 10.1097/RHU.0000000000000610

[8] Costello ME Elewaut D Kenna TJ: Microbes, the gut and ankylosing spondylitis. *Arthritis Res Ther.* 2013;15(3):214. 10.1186/ar4228 23750937 PMC4060176

[9] Benjamin R Parham P: HLA-B27 and disease: a consequence of inadvertent antigen presentation? *Rheum Dis Clin North Am.* 1992;18(1):11–21. 1561398

[10] Mear JP Schreiber KL Münz C: Misfolding of HLA-B27 as a result of its B pocket suggests a novel mechanism for its role in susceptibility to spondyloarthropathies. *J Immunol.* 1999;163(12):6665–70. 10586062

[11] Smith JA Colbert RA: Review: The interleukin-23/interleukin-17 axis in spondyloarthritis pathogenesis: Th17 and beyond. *Arthritis Rheumatol.* 2014;66(2):231–41. 10.1002/art.38291 24504793 PMC4058712

[12] Schittenhelm RB Sian TC Wilmann PG: Revisiting the arthritogenic peptide theory: quantitative not qualitative changes in the peptide repertoire of HLA-B27 allotypes. *Arthritis Rheumatol.* 2015;67(3):702–13. 10.1002/art.38963 25418920

[13] Allen RL O'Callaghan CA McMichael AJ: Cutting edge: HLA-B27 can form a novel beta 2-microglobulin-free heavy chain homodimer structure. *J Immunol.* 1999;162(9):5045–8. 10227970

[14] Kollnberger S Bird L Sun MY: Cell-surface expression and immune receptor recognition of HLA-B27 homodimers. *Arthritis Rheum.* 2002;46(11):2972–82. 10.1002/art.10605 12428240

[15] Kollnberger S Chan A Sun MY: Interaction of HLA-B27 homodimers with KIR3DL1 and KIR3DL2, unlike HLA-B27 heterotrimers, is independent of the sequence of bound peptide. *Eur J Immunol.* 2007;37(5):1313–22. 10.1002/eji.200635997 17407096

[16] Payeli SK Kollnberger S Marroquin Belaunzaran O: Inhibiting HLA-B27 homodimer-driven immune cell inflammation in spondylarthritis. *Arthritis Rheum.* 2012;64(10):3139–49. 10.1002/art.34538 22576154

[17] Ridley A Hatano H Wong-Baeza I: Activation-Induced Killer Cell Immunoglobulin-like Receptor 3DL2 Binding to HLA-B27 Licenses Pathogenic T Cell Differentiation in Spondyloarthritis. *Arthritis Rheumatol.* 2016;68(4):901–14. 10.1002/art.39515 26841353 PMC4855641

[18] Dong C: T _H_17 cells in development: an updated view of their molecular identity and genetic programming. *Nat Rev Immunol.* 2008;8(5):337–48. 10.1038/nri2295 18408735

[19] Yen D Cheung J Scheerens H: IL-23 is essential for T cell-mediated colitis and promotes inflammation via IL-17 and IL-6. *J Clin Invest.* 2006;116(5):1310–6. 10.1172/JCI21404 16670770 PMC1451201

[20] Oppmann B Lesley R Blom B: Novel p19 protein engages IL-12p40 to form a cytokine, IL-23, with biological activities similar as well as distinct from IL-12. *Immunity.* 2000;13(5):715–25. 10.1016/S1074-7613(00)00070-4 11114383

[21] Jain R Chen Y Kanno Y: Interleukin-23-Induced Transcription Factor Blimp-1 Promotes Pathogenicity of T Helper 17 Cells. *Immunity.* 2016;44(1):131–42. 10.1016/j.immuni.2015.11.009 26750311 PMC11608061

[22] Ellinghaus D Zhang H Zeissig S: Association between variants of *PRDM1* and *NDP52* and Crohn's disease, based on exome sequencing and functional studies. *Gastroenterology.* 2013;145(2):339–47. 10.1053/j.gastro.2013.04.040 23624108 PMC3753067

[23] Ghoreschi K Laurence A Yang XP: Generation of pathogenic T _H_17 cells in the absence of TGF-β signalling. *Nature.* 2010;467(7318):967–71. 10.1038/nature09447 20962846 PMC3108066

[24] Gagliani N Amezcua Vesely MC Iseppon A: Th17 cells transdifferentiate into regulatory T cells during resolution of inflammation. *Nature.* 2015;523(7559):221–5. 10.1038/nature14452 25924064 PMC4498984

[25] Maxwell JR Zhang Y Brown WA: Differential Roles for Interleukin-23 and Interleukin-17 in Intestinal Immunoregulation. *Immunity.* 2015;43(4):739–50. 10.1016/j.immuni.2015.08.019 26431947

[26] Ohnmacht C Park JH Cording S: MUCOSAL IMMUNOLOGY. The microbiota regulates type 2 immunity through RORγt ^+^ T cells. *Science.* 2015;349(6251):989–93. 10.1126/science.aac4263 26160380

[27] Fasching P Stradner M Graninger W: Therapeutic Potential of Targeting the Th17/Treg Axis in Autoimmune Disorders. *Molecules.* 2017;22(1). pii: E134. 10.3390/molecules22010134 28098832 PMC6155880

[28] Costello ME Ciccia F Willner D: Brief Report: Intestinal Dysbiosis in Ankylosing Spondylitis. *Arthritis Rheumatol.* 2015;67(3):686–91. 10.1002/art.38967 25417597

[29] Ciccia F Rizzo A Triolo G: Subclinical gut inflammation in ankylosing spondylitis. *Curr Opin Rheumatol.* 2016;28(1):89–96. 10.1097/BOR.0000000000000239 26599385

[30] Jacques P Elewaut D Mielants H: Interactions between gut inflammation and arthritis/spondylitis. *Curr Opin Rheumatol.* 2010;22(4):368–74. 10.1097/BOR.0b013e3283393807 20485176

[31] Van Praet L Van den Bosch FE Jacques P: Microscopic gut inflammation in axial spondyloarthritis: a multiparametric predictive model. *Ann Rheum Dis.* 2013;72(3):414–7. 10.1136/annrheumdis-2012-202135 23139267

[32] Asquith M Elewaut D Lin P: The role of the gut and microbes in the pathogenesis of spondyloarthritis. *Best Pract Res Clin Rheumatol.* 2014;28(5):687–702. 10.1016/j.berh.2014.10.018 25488778 PMC4293151

[33] Dalal SR Chang EB: The microbial basis of inflammatory bowel diseases. *J Clin Invest.* 2014;124(10):4190–6. 10.1172/JCI72330 25083986 PMC4191005

[34] Martínez-González O Cantero-Hinojosa J Paule-Sastre P: Intestinal permeability in patients with ankylosing spondylitis and their healthy relatives. *Br J Rheumatol.* 1994;33(7):644–7. 10.1093/rheumatology/33.7.644 8019793

[35] Taurog JD Richardson JA Croft JT: The germfree state prevents development of gut and joint inflammatory disease in HLA-B27 transgenic rats. *J Exp Med.* 1994;180(6):2359–64. 10.1084/jem.180.6.2359 7964509 PMC2191772

[36] Ebringer A: The relationship between *Klebsiella* infection and ankylosing spondylitis. *Baillieres Clin Rheumatol.* 1989;3(2):321–38. 10.1016/S0950-3579(89)80024-X 2670258

[37] Rosenbaum JT Asquith MJ: The Microbiome: a Revolution in Treatment for Rheumatic Diseases? *Curr Rheumatol Rep.* 2016;18(10):62. 10.1007/s11926-016-0614-8 27641915

[38] Kang EH Lee JT Lee HJ: Chronic Periodontitis Is Associated With Spinal Dysmobility in Patients With Ankylosing Spondylitis. *J Periodontol.* 2015;86(12):1303–13. 10.1902/jop.2015.150202 26291296

[39] Bautista-Molano W van der Heijde D Landewé R: Is there a relationship between spondyloarthritis and periodontitis? A case-control study. *RMD Open.* 2017;3(2):e000547. 10.1136/rmdopen-2017-000547 29299339 PMC5729302

[40] Landi M Maldonado-Ficco H Perez-Alamino R: Gender differences among patients with primary ankylosing spondylitis and spondylitis associated with psoriasis and inflammatory bowel disease in an iberoamerican spondyloarthritis cohort. *Medicine (Baltimore).* 2016;95(51):e5652. 10.1097/MD.0000000000005652 28002334 PMC5181818

[41] Rusman T van Vollenhoven RF van der Horst-Bruinsma IE: Gender Differences in Axial Spondyloarthritis: Women Are Not So Lucky. *Curr Rheumatol Rep.* 2018;20(6):35. 10.1007/s11926-018-0744-2 29754330 PMC5949138

[42] Webers C Essers I Ramiro S: Gender-attributable differences in outcome of ankylosing spondylitis: long-term results from the Outcome in Ankylosing Spondylitis International Study. *Rheumatology (Oxford).* 2016;55(3):419–28. 10.1093/rheumatology/kev340 26385369

[43] Park W Yoo DH Jaworski J: Comparable long-term efficacy, as assessed by patient-reported outcomes, safety and pharmacokinetics, of CT-P13 and reference infliximab in patients with ankylosing spondylitis: 54-week results from the randomized, parallel-group PLANETAS study. *Arthritis Res Ther.* 2016;18:25. 10.1186/s13075-016-0930-4 26795209 PMC4721187

[44] Emery P Vencovský J Sylwestrzak A: 52-week results of the phase 3 randomized study comparing SB4 with reference etanercept in patients with active rheumatoid arthritis. *Rheumatology (Oxford).* 2017;56(12):2093–101. 10.1093/rheumatology/kex269 28968793 PMC5850652

[45] Braun J Baraliakos X Deodhar A: Effect of secukinumab on clinical and radiographic outcomes in ankylosing spondylitis: 2-year results from the randomised phase III MEASURE 1 study. *Ann Rheum Dis.* 2017;76(6):1070–7. 10.1136/annrheumdis-2016-209730 27965257

[46] https://clinicaltrials.gov/ct2/show/NCT02696785.

[47] https://clinicaltrials.gov/ct2/show/study/NCT02429882.

[48] https://clinicaltrials.gov/ct2/show/NCT02438787.

[49] https://clinicaltrials.gov/ct2/show/NCT02437162.

[50] Genovese MC Weinblatt ME Mease PJ: Dual inhibition of tumour necrosis factor and interleukin-17A with ABT-122: open-label long-term extension studies in rheumatoid arthritis or psoriatic arthritis. *Rheumatology (Oxford).* 2018. 10.1093/rheumatology/key173 30032191

[51] https://clinicaltrials.gov/ct2/show/NCT02243787.

[52] Hammitzsch A Tallant C Fedorov O: CBP30, a selective CBP/p300 bromodomain inhibitor, suppresses human Th17 responses. *Proc Natl Acad Sci U S A.* 2015;112(34):10768–73. 10.1073/pnas.1501956112 26261308 PMC4553799

[53] Sieper J Braun J Kay J: Sarilumab for the treatment of ankylosing spondylitis: Results of a Phase II, randomised, double-blind, placebo-controlled study (ALIGN). *Ann Rheum Dis.* 2015;74(6):1051–7. 10.1136/annrheumdis-2013-204963 24550171 PMC4431338

[54] van der Heijde D Deodhar A Wei JC: Tofacitinib in patients with ankylosing spondylitis: a phase II, 16-week, randomised, placebo-controlled, dose-ranging study. *Ann Rheum Dis.* 2017;76(8):1340–7. 10.1136/annrheumdis-2016-210322 28130206 PMC5738601

[55] Dougados M Sepriano A Molto A: Sacroiliac radiographic progression in recent onset axial spondyloarthritis: the 5-year data of the DESIR cohort. *Ann Rheum Dis.* 2017;76(11):1823–8. 10.1136/annrheumdis-2017-211596 28684556 PMC5705846

[56] Cho IH Lee N Song D: Evaluation of the structural, physicochemical, and biological characteristics of SB4, a biosimilar of etanercept. *MAbs.* 2016;8(6):1136–55. 10.1080/19420862.2016.1193659 27246928 PMC4968139

[57] Park W Hrycaj P Jeka S: A randomised, double-blind, multicentre, parallel-group, prospective study comparing the pharmacokinetics, safety, and efficacy of CT-P13 and innovator infliximab in patients with ankylosing spondylitis: the PLANETAS study. *Ann Rheum Dis.* 2013;72(10):1605–12. 10.1136/annrheumdis-2012-203091 23687259 PMC3786643

[58] Park W Yoo DH Miranda P: Efficacy and safety of switching from reference infliximab to CT-P13 compared with maintenance of CT-P13 in ankylosing spondylitis: 102-week data from the PLANETAS extension study. *Ann Rheum Dis.* 2017;76(2):346–54. 10.1136/annrheumdis-2015-208783 27117698 PMC5284340

[59] Yoo DH Racewicz A Brzezicki J: A phase III randomized study to evaluate the efficacy and safety of CT-P13 compared with reference infliximab in patients with active rheumatoid arthritis: 54-week results from the PLANETRA study. *Arthritis Res Ther.* 2016;18:82. 10.1186/s13075-016-0981-6 27038608 PMC4818886

[60] Yoo DH Prodanovic N Jaworski J: Efficacy and safety of CT-P13 (biosimilar infliximab) in patients with rheumatoid arthritis: comparison between switching from reference infliximab to CT-P13 and continuing CT-P13 in the PLANETRA extension study. *Ann Rheum Dis.* 2017;76(2):355–63. 10.1136/annrheumdis-2015-208786 27130908 PMC5284338

[61] Emery P Vencovský J Sylwestrzak A: Long-term efficacy and safety in patients with rheumatoid arthritis continuing on SB4 or switching from reference etanercept to SB4. *Ann Rheum Dis.* 2017; pii: annrheumdis-2017-211591. 10.1136/annrheumdis-2017-211591 28794078 PMC5705842

[62] https://clinicaltrials.gov/ct2/show/NCT03100734.

[63] Liu W Wu YH Zhang L: Elevated serum levels of IL-6 and IL-17 may associate with the development of ankylosing spondylitis. *Int J Clin Exp Med.* 2015;8(10):17362–76. 26770328 PMC4694228

[64] Yago T Nanke Y Kawamoto M: IL-23 and Th17 Disease in Inflammatory Arthritis. *J Clin Med.* 2017;6(9): pii: E81. 10.3390/jcm6090081 28850053 PMC5615274

[65] Baraliakos X Kivitz AJ Deodhar AA: Long-term effects of interleukin-17A inhibition with secukinumab in active ankylosing spondylitis: 3-year efficacy and safety results from an extension of the Phase 3 MEASURE 1 trial. *Clin Exp Rheumatol.* 2018;36(1):50–5. 28516874

[66] Marzo-Ortega H Sieper J Kivitz A: Secukinumab and Sustained Improvement in Signs and Symptoms of Patients With Active Ankylosing Spondylitis Through Two Years: Results From a Phase III Study. *Arthritis Care Res (Hoboken).* 2017;69(7):1020–9. 10.1002/acr.23233 28235249 PMC5518281

[67] Pavelka K Kivitz A Dokoupilova E: Efficacy, safety, and tolerability of secukinumab in patients with active ankylosing spondylitis: a randomized, double-blind phase 3 study, MEASURE 3. *Arthritis Res Ther.* 2017;19(1):285. 10.1186/s13075-017-1490-y 29273067 PMC5741872

[68] https://www.clinicaltrialsregister.eu/ctr-search/trial/2015-005021-39/CZ.

[69] https://clinicaltrials.gov/ct2/show/study/NCT02696798.

[70] Sherlock JP Joyce-Shaikh B Turner SP: IL-23 induces spondyloarthropathy by acting on ROR-γt ^+^ CD3 ^+^CD4 ^-^CD8 ^-^ entheseal resident T cells. *Nat Med.* 2012;18(7):1069–76. 10.1038/nm.2817 22772566

[71] McInnes IB Kavanaugh A Gottlieb AB: Efficacy and safety of ustekinumab in patients with active psoriatic arthritis: 1 year results of the phase 3, multicentre, double-blind, placebo-controlled PSUMMIT 1 trial. *Lancet.* 2013;382(9894):780–9. 10.1016/S0140-6736(13)60594-2 23769296

[72] Poddubnyy D Hermann KG Callhoff J: Ustekinumab for the treatment of patients with active ankylosing spondylitis: results of a 28-week, prospective, open-label, proof-of-concept study (TOPAS). *Ann Rheum Dis.* 2014;73(5):817–23. 10.1136/annrheumdis-2013-204248 24389297

[73] Fleischmann RM Wagner F Kivitz AJ: Safety, Tolerability, and Pharmacodynamics of ABT-122, a Tumor Necrosis Factor- and Interleukin-17-Targeted Dual Variable Domain Immunoglobulin, in Patients With Rheumatoid Arthritis. *Arthritis Rheumatol.* 2017;69(12):2283–91. 10.1002/art.40319 28941216

[74] Silacci M Lembke W Woods R: Discovery and characterization of COVA322, a clinical-stage bispecific TNF/IL-17A inhibitor for the treatment of inflammatory diseases. *MAbs.* 2016;8(1):141–9. 10.1080/19420862.2015.1093266 26390837 PMC4966518

[75] van der Heijde D Ramiro S Landewé R: 2016 update of the ASAS-EULAR management recommendations for axial spondyloarthritis. *Ann Rheum Dis.* 2017;76(6):978–91. 10.1136/annrheumdis-2016-210770 28087505

[76] Závada J Uher M Sisol K: A tailored approach to reduce dose of anti-TNF drugs may be equally effective, but substantially less costly than standard dosing in patients with ankylosing spondylitis over 1 year: a propensity score-matched cohort study. *Ann Rheum Dis.* 2016;75(1):96–102. 10.1136/annrheumdis-2014-205202 25165033

[77] Plasencia C Kneepkens EL Wolbink G: Comparing Tapering Strategy to Standard Dosing Regimen of Tumor Necrosis Factor Inhibitors in Patients with Spondyloarthritis in Low Disease Activity. *J Rheumatol.* 2015;42(9):1638–46. 10.3899/jrheum.141128 26178279

[78] Park JW Kwon HM Park JK: Impact of Dose Tapering of Tumor Necrosis Factor Inhibitor on Radiographic Progression in Ankylosing Spondylitis. *PLoS One.* 2016;11(12):e0168958. 10.1371/journal.pone.0168958 28033420 PMC5199008

[79] Fattahi MJ Jamshidi AR Mahmoudi M: Evaluation of the efficacy and safety of β-d-mannuronic acid in patients with ankylosing spondylitis: A 12-week randomized, placebo-controlled, phase I/II clinical trial. *Int Immunopharmacol.* 2018;54:112–7. 10.1016/j.intimp.2017.11.003 29127910

[80] Wanders A Heijde Dv Landewé R: Nonsteroidal antiinflammatory drugs reduce radiographic progression in patients with ankylosing spondylitis: a randomized clinical trial. *Arthritis Rheum.* 2005;52(6):1756–65. 10.1002/art.21054 15934081

[81] Sieper J Listing J Poddubnyy D: Effect of continuous versus on-demand treatment of ankylosing spondylitis with diclofenac over 2 years on radiographic progression of the spine: results from a randomised multicentre trial (ENRADAS). *Ann Rheum Dis.* 2016;75(8):1438–43. 10.1136/annrheumdis-2015-207897 26242443

[82] Spoorenberg A van der Heijde D de Klerk E: Relative value of erythrocyte sedimentation rate and C-reactive protein in assessment of disease activity in ankylosing spondylitis. *J Rheumatol.* 1999;26(4):980–4. 10229432

[83] Wallis D Haroon N Ayearst R: Ankylosing spondylitis and nonradiographic axial spondyloarthritis: part of a common spectrum or distinct diseases? *J Rheumatol.* 2013;40(12):2038–41. 10.3899/jrheum.130588 24187102

[84] Gratacós J Collado A Filella X: Serum cytokines (IL-6, TNF-alpha, IL-1 beta and IFN-gamma) in ankylosing spondylitis: a close correlation between serum IL-6 and disease activity and severity. *Br J Rheumatol.* 1994;33(10):927–31. 10.1093/rheumatology/33.10.927 7921752

[85] Bal A Unlu E Bahar G: Comparison of serum IL-1 beta, sIL-2R, IL-6, and TNF-alpha levels with disease activity parameters in ankylosing spondylitis. *Clin Rheumatol.* 2007;26(2):211–5. 10.1007/s10067-006-0283-5 16583185

[86] Romero-Sanchez C Jaimes DA Londoño J: Association between Th-17 cytokine profile and clinical features in patients with spondyloarthritis. *Clin Exp Rheumatol.* 2011;29(5):828–34. 22041179

[87] Pedersen SJ Sørensen IJ Garnero P: ASDAS, BASDAI and different treatment responses and their relation to biomarkers of inflammation, cartilage and bone turnover in patients with axial spondyloarthritis treated with TNFα inhibitors. *Ann Rheum Dis.* 2011;70(8):1375–81. 10.1136/ard.2010.138883 21551511

[88] Sveaas SH Berg IJ Provan SA: Circulating levels of inflammatory cytokines and cytokine receptors in patients with ankylosing spondylitis: a cross-sectional comparative study. *Scand J Rheumatol.* 2015;44(2):118–24. 10.3109/03009742.2014.956142 25756521

[89] Lee YW Lee KM Lee JM: The usefulness of fecal calprotectin in assessing inflammatory bowel disease activity. *Korean J Intern Med.* 2018. 10.3904/kjim.2016.324 29347813 PMC6325438

[90] Duran A Kobak S Sen N: Fecal calprotectin is associated with disease activity in patients with ankylosing spondylitis. *Bosn J Basic Med Sci.* 2016;16(1):71–4. 10.17305/bjbms.2016.752 26773186 PMC4765943

[91] Klingberg E Oleröd G Hammarsten O: The vitamin D status in ankylosing spondylitis in relation to intestinal inflammation, disease activity, and bone health: a cross-sectional study. *Osteoporos Int.* 2016;27(6):2027–33. 10.1007/s00198-016-3489-7 26809190

[92] Levitova A Hulejova H Spiritovic M: Clinical improvement and reduction in serum calprotectin levels after an intensive exercise programme for patients with ankylosing spondylitis and non-radiographic axial spondyloarthritis. *Arthritis Res Ther.* 2016;18(1):275. 10.1186/s13075-016-1180-1 27887637 PMC5124318

[93] Rossini M Viapiana O Adami S: Focal bone involvement in inflammatory arthritis: the role of IL17. *Rheumatol Int.* 2016;36(4):469–82. 10.1007/s00296-015-3387-x 26521079

[94] Chabaud M Miossec P: The combination of tumor necrosis factor alpha blockade with interleukin-1 and interleukin-17 blockade is more effective for controlling synovial inflammation and bone resorption in an *ex vivo* model. *Arthritis Rheum.* 2001;44(6):1293–303. 10.1002/1529-0131(200106)44:6<1293::AID-ART221>3.0.CO;2-T 11407688

[95] Lubberts E Joosten LA Oppers B: IL-1-independent role of IL-17 in synovial inflammation and joint destruction during collagen-induced arthritis. *J Immunol.* 2001;167(2):1004–13. 10.4049/jimmunol.167.2.1004 11441109

[96] Sato K Suematsu A Okamoto K: Th17 functions as an osteoclastogenic helper T cell subset that links T cell activation and bone destruction. *J Exp Med.* 2006;203(12):2673–82. 10.1084/jem.20061775 17088434 PMC2118166

[97] Baron R Rawadi G: Targeting the Wnt/beta-catenin pathway to regulate bone formation in the adult skeleton. *Endocrinology.* 2007;148(6):2635–43. 10.1210/en.2007-0270 17395698

[98] Glass DA 2nd Bialek P Ahn JD: Canonical Wnt signaling in differentiated osteoblasts controls osteoclast differentiation. *Dev Cell.* 2005;8(5):751–64. 10.1016/j.devcel.2005.02.017 15866165

[99] Osta B Lavocat F Eljaafari A: Effects of Interleukin-17A on Osteogenic Differentiation of Isolated Human Mesenchymal Stem Cells. *Front Immunol.* 2014;5:425. 10.3389/fimmu.2014.00425 25228904 PMC4151036

[100] Zhang F Wang CL Koyama Y: Compressive force stimulates the gene expression of IL-17s and their receptors in MC3T3-E1 cells. *Connect Tissue Res.* 2010;51(5):359–69. 10.3109/03008200903456942 20497006

[101] Perpétuo IP Caetano-Lopes J Vieira-Sousa E: Ankylosing Spondylitis Patients Have Impaired Osteoclast Gene Expression in Circulating Osteoclast Precursors. *Front Med (Lausanne).* 2017;4:5. 10.3389/fmed.2017.00005 28191455 PMC5269449

[102] Ranganathan V Ciccia F Zeng F: Macrophage Migration Inhibitory Factor Induces Inflammation and Predicts Spinal Progression in Ankylosing Spondylitis. *Arthritis Rheumatol.* 2017;69(9):1796–806. 10.1002/art.40175 28597514

[103] El-Zayadi AA Jones EA Churchman SM: Interleukin-22 drives the proliferation, migration and osteogenic differentiation of mesenchymal stem cells: a novel cytokine that could contribute to new bone formation in spondyloarthropathies. *Rheumatology (Oxford).* 2017;56(3):488–93. 10.1093/rheumatology/kew384 27940584

[104] Lee EJ Lee EJ Chung YH: High level of interleukin-32 gamma in the joint of ankylosing spondylitis is associated with osteoblast differentiation. *Arthritis Res Ther.* 2015;17:350. 10.1186/s13075-015-0870-4 26634249 PMC4669668

[105] Fawzy RM Ganeb SS Said EA: Serum Level of Interleukin-37 and Expression of Its mRNA in Ankylosing Spondylitis Patients: Possible Role in Osteoporosis. *Egypt J Immunol.* 2016;23(1):19–29. 28502149

[106] Kwon SR Jung KH Lim MJ: The effect of anti-TNF treatment on osteoblastogenesis in ankylosing spondylitis: the number of circulating osteoblast-lineage cells in peripheral blood decreased after infliximab therapy in patients with ankylosing spondylitis. *Clin Exp Rheumatol.* 2017;35(5):837–43. 28375831

[107] Machado PM Baraliakos X van der Heijde D: MRI vertebral corner inflammation followed by fat deposition is the strongest contributor to the development of new bone at the same vertebral corner: a multilevel longitudinal analysis in patients with ankylosing spondylitis. *Ann Rheum Dis.* 2016;75(8):1486–93. 10.1136/annrheumdis-2015-208011 26462728

[108] Wanders AJ Landewé RB Spoorenberg A: What is the most appropriate radiologic scoring method for ankylosing spondylitis? A comparison of the available methods based on the Outcome Measures in Rheumatology Clinical Trials filter. *Arthritis Rheum.* 2004;50(8):2622–32. 10.1002/art.20446 15334477

[109] Kim JY Lee S Joo KB: Loss of anterior concavity of the first sacrum can predict spinal involvement in ankylosing spondylitis. *Rheumatol Int.* 2016;36(1):161–5. 10.1007/s00296-015-3359-1 26387092

[110] Ralston SH Urquhart GD Brzeski M: A new method for the radiological assessment of vertebral squaring in ankylosing spondylitis. *Ann Rheum Dis.* 1992;51(3):330–3. 10.1136/ard.51.3.330 1575575 PMC1004654

[111] Munk HL Gudmann NS Christensen AF: Cartilage collagen type II seromarker patterns in axial spondyloarthritis and psoriatic arthritis: associations with disease activity, smoking and HLA-B _27_. *Rheumatol Int.* 2016;36(4):541–9. 10.1007/s00296-015-3397-8 26620690

[112] Althoff CE Feist E Burova E: Magnetic resonance imaging of active sacroiliitis: do we really need gadolinium? *Eur J Radiol.* 2009;71(2):232–6. 10.1016/j.ejrad.2009.04.034 19446974

[113] Zhao YH Cao YY Zhang Q: Role of Diffusion-weighted and Contrast-enhanced Magnetic Resonance Imaging in Differentiating Activity of Ankylosing Spondylitis. *Chin Med J (Engl).* 2017;130(11):1303–8. 10.4103/0366-6999.206359 28524829 PMC5455039

[114] Bradbury LA Hollis KA Gautier B: Diffusion-weighted Imaging Is a Sensitive and Specific Magnetic Resonance Sequence in the Diagnosis of Ankylosing Spondylitis. *J Rheumatol.* 2018;45(6):771–8. 10.3899/jrheum.170312 29449501

[115] Weber U Pedersen SJ Østergaard M: Can erosions on MRI of the sacroiliac joints be reliably detected in patients with ankylosing spondylitis? - A cross-sectional study. *Arthritis Res Ther.* 2012;14(3):R124. 10.1186/ar3854 22626458 PMC3446505

[116] Laloo F Herregods N Varkas G: MR signal in the sacroiliac joint space in spondyloarthritis: a new sign. *Eur Radiol.* 2017;27(5):2024–30. 10.1007/s00330-016-4587-9 27651143

[117] Hu Z Wang X Qi J: Backfill is a specific sign of axial spondyloarthritis seen on MRI. *Joint Bone Spine.* 2016;83(2):179–83. 10.1016/j.jbspin.2015.05.011 26709251

